# Dual Mode of the Saponin Aescin in Plant Protection: Antifungal Agent and Plant Defense Elicitor

**DOI:** 10.3389/fpls.2019.01448

**Published:** 2019-11-28

**Authors:** Lucie Trdá, Martin Janda, Denisa Macková, Romana Pospíchalová, Petre I. Dobrev, Lenka Burketová, Pavel Matušinsky

**Affiliations:** ^1^Laboratory of Pathological Plant Physiology, Institute of Experimental Botany of The Czech Academy of Sciences, Prague, Czechia; ^2^Laboratory of Plant Biochemistry, Department of Biochemistry and Microbiology, University of Chemistry and Technology Prague, Prague, Czechia; ^3^Department Genetics, Faculty of Biology, Biocenter, Ludwig-Maximilian-University of Munich (LMU), Martinsried, Germany; ^4^Laboratory of Hormonal Regulations in Plants, Institute of Experimental Botany of The Czech Academy of Sciences, Prague, Czechia; ^5^Department of Plant Pathology, Agrotest Fyto, Ltd, Kroměrˇíž, Czechia; ^6^Department of Botany, Faculty of Science, Palacký University in Olomouc, Olomouc, Czechia

**Keywords:** Brassica napus, *Leptosphaeria maculans*, salicylic acid, fungicide, *Pseudomonas syringae*, *Arabidopsis thaliana*, EC_50_

## Abstract

Being natural plant antimicrobials, saponins have potential for use as biopesticides. Nevertheless, their activity in plant–pathogen interaction is poorly understood. We performed a comparative study of saponins' antifungal activities on important crop pathogens based on their effective dose (EC_50_) values. Among those saponins tested, aescin showed itself to be the strongest antifungal agent. The antifungal effect of aescin could be reversed by ergosterol, thus suggesting that aescin interferes with fungal sterols. We tested the effect of aescin on plant–pathogen interaction in two different pathosystems: *Brassica napus* versus (fungus) *Leptosphaeria maculans* and *Arabidopsis thaliana* versus (bacterium) *Pseudomonas syringae* pv *tomato* DC3000 (*Pst* DC3000). We analyzed resistance assays, defense gene transcription, phytohormonal production, and reactive oxygen species production. Aescin activated *B. napus* defense through induction of the salicylic acid pathway and oxidative burst. This defense response led finally to highly efficient plant protection against *L. maculans* that was comparable to the effect of fungicides. Aescin also inhibited colonization of *A. thaliana* by *Pst* DC3000, the effect being based on active elicitation of salicylic acid (SA)-dependent immune mechanisms and without any direct antibacterial effect detected. Therefore, this study brings the first report on the ability of saponins to trigger plant immune responses. Taken together, aescin in addition to its antifungal properties activates plant immunity in two different plant species and provides SA-dependent resistance against both fungal and bacterial pathogens.

## Introduction

Crop production is hampered by numerous plant diseases caused by diverse pathogenic microorganisms, such as fungi, bacteria or pests, affecting yield, harvest quality and safety. Although pesticides are currently employed to control crop pathogens and pests, growing problems of fungal resistance to fungicides appear to pose a serious future threat to agriculture (Fisher et al., 2018). Moreover, alternatives to fungicides are needed that are less harmful to health and the environment. These might include more intensive employment of biological control, greater crop diversity (Zhu et al., 2000), or developing safer compounds with new modes of action (Burketová et al., 2015). Higher plants could constitute a great source of such compounds. Most plants produce a wide variety of antimicrobial secondary metabolites, including alkaloids, flavonoids, terpenes, organic acids, essential oils, and saponins that are involved in plant defense responses essential for plant protection against microbial or pest attack (Osbourn, 1996; Field et al., 2006; da Cruz Cabral et al., 2013; Matušinský et al., 2015).

Saponins occur in a wide range of plant species (Price et al., 1987; Moses et al., 2014). They comprise a structurally diverse family of triterpenoids, steroids or steroidal glycoalkaloids (Podolak et al., 2010; Moses et al., 2014). Saponins exhibit amphiphilic properties that are due to the linkage of a lipophilic triterpene derivative (sapogenin) to one or more hydrophilic glycoside moieties. Historically, plant extracts from *Saponaria officinalis* have been used for their soap properties (Hostettmann and Marston, 1995). Saponins have a broad spectrum of activities in living organisms. They are generally antimicrobial against bacteria and fungi invading plants (Gruiz, 1996; Zablotowicz et al., 1996; Papadopoulou et al., 1999; Barile et al., 2007; Hoagland, 2009; Moses et al., 2014), but they were also effectively applied against microbes associated with animals (Yang et al., 2006; Saleem et al., 2010). Furthermore, saponins exert insecticidal (Nielsen et al., 2010; Singh and Kaur, 2018), antiviral (Zhao et al., 2008), and molluscicidal (Huang et al., 2003) activities, as well as allelopathic activity towards other plant species (Waller et al., 1993).

Saponins are mainly considered to comprise a part of plants' antimicrobial defense system. The underlying mechanisms of their activity are understood to be based on their ability to form complexes with sterols present in the membrane of microorganisms and consequently to cause membrane perturbation (Steel and Drysdale, 1988; Morrissey and Osbourn, 1999; Augustin et al., 2011; Sreij et al., 2019). The antifungal activity of saponins has been known for decades (Turner, 1960; Wolters, 1966; Gruiz, 1996) and their activity against fungal plant pathogens of crops has been reported previously. For example, minutoside saponins and sapogenins, alliogenin, and neoagigenin, isolated from the bulbs of *Allium minutiflorum* showed antimicrobial activity against various soil-borne and air-borne fungal pathogens (Barile et al., 2007). Saponin alliospiroside extracted from *Allium cepa* protected strawberry plants against *Colletotrichum gloeosporioides*, thus indicating a potential to control anthracnose of the plant (Teshima et al., 2013). To date, however, only limited work has been reported toward quantifying antifungal activity against phytopathogenic fungi by establishing EC_50_ values (Saniewska et al., 2006; Porsche et al., 2018), and parallel comparisons with fungicides are often lacking. Moreover, effects on plants have been tested only by several studies (Hoagland et al., 1996; Hoagland, 2009). The goal of the present study was to investigate the potential of plant saponins as an alternative to fungicide treatment on crops.

We focus here mainly on the pathosystem of the crop *Brassica napus* (oilseed rape) and its devastating fungal hemibiotrophic pathogen *Leptosphaeria maculans*, an infectious agent of phoma stem canker in oilseed rape. Plants face microbial infections through an efficient immune system. Plant immunity is very complex, consisting of pathogen recognition by plant immune receptors, signaling events, such as reactive oxygen species (ROS) production or MAP kinase activation, which ultimately triggers such defense mechanisms as changes in gene transcription resulting in expression of antimicrobial proteins, phytohormone production, or callose accumulation (Dodds and Rathjen, 2010; Cook et al., 2015; Trdá et al., 2015). Signaling pathways of phytohormones, such as salicylic acid (SA), jasmonic acid (JA) or ethylene (ET) cross-communicate allowing the plant to finely regulate its immune responses (Glazebrook, 2005; Pieterse et al., 2009). Immune responses have been previously studied in *B. napus* (Šašek et al., 2012a; Šašek et al., 2012b; Lloyd et al., 2014; Nováková et al., 2014).

Plant treatment with diverse agents, including microbe-derived compounds, phytohormones and synthetic chemicals, can induce resistance to subsequent pathogen invasion both locally and systemically (Walters et al., 2013, Burketová et al., 2015). Such resistance, called systemic acquired resistance (SAR), is among others mediated and dependent on SA. SAR was inhibited in *npr1* or *ics1* mutant plants (Kachroo and Robin, 2013). SAR-inducing chemicals are employed in pest control. Benzothiadiazole (BTH) is a functional analog of salicylic acid (SA) and a synthetic inducer of resistance to pathogens (Friedrich et al., 1996; Walters et al., 2013). BTH activates the *B. napus* immune system and provides protection against *L. maculans* (Šašek et al., 2012a). We have previously shown that the phytohormone salicylic acid (SA) plays an important role upon *L. maculans* infection (Šašek et al., 2012b). SA's role in plant immunity is well established (Tsuda et al., 2013; Janda and Ruelland, 2015). Although SA can be involved also in response to some necrotrophic pathogens (Nováková et al., 2014), it is mostly connected with defense against biotrophic microorganisms (Glazebrook, 2005). Substantial knowledge about SA's role in plant disease resistance comes from studies using a model pathosystem involving *A. thaliana* and the bacteria *Pseudomonas syringae* pv tomato DC3000 (*Pst* DC3000) (Katagiri et al., 2002; Xin and He, 2013; Xin et al., 2018; Leontovyčová et al., 2019).

Here, we present a comprehensive and comparative study of antifungal activities against crop pathogens of three terpenoid saponins in comparison to fungicides in commercial use. We chose aescin as the best antifungal agent and further characterized its activity in plants. We show that aescin triggers plant defense by activating the SA pathway and oxidative burst, ultimately leading to highly efficient resistance of *B. napus* against the fungus *L. maculans*. The level of protection it provides is comparable to that of fungicides. In *A. thaliana*, aescin induces SA-dependent resistance to *Pst* DC3000. Therefore, we provide here evidence of aescin's dual mode of action in plant defense.

## Material and Methods

### Fungal Isolates and Cultivation

Fungal isolates (with the exception of *L. maculans* JN2) were acquired in the territory of the Czech Republic from symptomatic crop tissue in the field during the period 2002–2015. *Microdochium nivale* (Mn177 and Mn30) and *Oculimacula yallundae* (Oy19 and Oy221) were isolated from the stem bases of wheat in 2013 (Matušinský et al., 2017). *Zymoseptoria tritici* (Zt88 and Zt96) was collected from the leaves of winter wheat in 2013, and *Fusarium culmorum* strains (Fc107 and Fc289) were collected from wheat grains after harvest in 2010 and 2002, respectively (Matušinský et al., 2015). *Pyrenophora teres* (Ptt52 and Ptt17) and *Ramularia collo-cygni* (Rcc11 and Rcc41) were collected from leaves of spring barley in 2013. *L. maculans* (Lm170, Lm1-Lm4) isolates were collected from leaves of oilseed rape during 2014–2015.

*Pyrenophora teres* and *R. collo-cygni* conidia were transferred from the symptomatic leaves to Petri dishes with potato dextrose agar (PDA) media containing 50 µg·ml^−1^ of ampicillin. Conidia were spread over the surface of media and cultivated for 24–96 h at 18°C. Single-spore microcolonies were transferred into new Petri dishes. In the case of *L. maculans*, a single pycnidium from a symptomatic leaf was transferred to a droplet of sterile water on a glass microscope slide. The pycnidium was crushed by a cover glass and a part of the conidia was spread using a sterile needle over a solid PDA medium. After 3 days at 18°C in darkness, single microcolonies were transferred to new PDA plates. The *L. maculans* isolate JN2, also referred to as v23.1.2 (Balesdent et al., 2001; Šašek et al., 2012b), was used for most of the assays. Conidia of isolates JN2 and JN2-sGFP (JN2 transformed using a pCAMBgfp construct (Šašek et al., 2012a) were obtained from sporulating mycelium 10 days old kept under a 14h/10h light/dark regime (150 µE·m^−2^·s^−1^, 22°C, 70% relative humidity) in a cultivation chamber as described by Šašek et al. (2012b). Conidia were stored in concentration 10^8^ conidia·ml^−1^ at −20°C for up to 6 months.

### Antifungal and Antibacterial Assays

The radial growth of fungal mycelium was analyzed on PDA plates using the agar dilution method. Mycelial discs, 2 mm in diameter, were cut from the margins of colonies 5 days old and transferred to medium supplemented with streptomycin sulfate (50 µg·ml^−1^) and saponins (0, 10, 25, 50, and 100 µg·ml^−1^). After incubation at 18°C in darkness for 3 days in cases of rapidly growing fungi (*F. culmorum*, *L. maculans*, *M. nivale*, and *P. teres*) and 14 days in case of slowly growing fungi (*O. yallundae*, *R. collo-cygni*, and *Z. tritici*), the colony diameters were measured and compared to control plates lacking a saponin. Each isolate was analyzed in four technical replicates (four mycelial discs per plate) and in three independent biological experiments.

The conidial growth of *L. maculans* JN2-GFP isolate was analyzed in Gamborg B5 medium (Duchefa) supplemented with 0.3% (w/v) sucrose and 10 mM MES buffer (pH 6.8) at the final concentration of 2500 conidia per well of black 96-well plate (Nunc^®^). Aescin was used in the concentration range 0–100 µg·ml^−1^. Plates were incubated in darkness at 26°C for 4 days. Fluorescence was measured in eight wells for each treatment using a Tecan F200 fluorescence reader (Tecan, Männedorf, Switzerland) with 485/20 nm excitation filter and 535/25 nm emission filter. For both assays, the final concentration of EtOH in all treatments was 1% (v/v). Effective dose (EC_50_) values were calculated by probit analysis (Finney, 1971) using Biostat software (AnalystSoft Inc., Walnut, CA, USA). For microscopic analysis, the content of each well was transferred to a microscopic slide and observed under a Leica DM 5000 B fluorescence microscope (Leica, Germany).

To monitor antibacterial activity of aescin, a fresh bacterial suspension (OD_600_ of 0.005) in liquid LB medium was prepared from *Pst* DC3000 culture grown overnight on LB agar plates. Aescin (10 µg·ml^−1^) or EtOH (0.1%) was added to this suspension and OD_600_ was measured after 24 h, with three independent samples being used for each treatment.

### Fungal Treatment for Gene Expression

For gene expression, 10^7^ conidia of JN2-GFP were grown in 100 ml of Gamborg B5 medium (Duchefa, G0210, Haarlem, The Netherlands) supplemented with 3% (w/v) sucrose and 10 mM MES (pH 6.8) in Erlenmeyer flasks. Cultures were kept at 26°C in darkness and at constant shaking of 130 rpm in an orbital shaker (JeioTech, Seoul, Korea). The culture at day 7 was treated in sterile conditions with aescin, fungicide, or control (EtOH). The concentration of EtOH solvent was identical in each treatment. Samples were collected after 24 hours of treatment and processed as described for plant samples.

### Plant Cultivation and Treatment

*Brassica napus* plants of cultivar (cv.) Columbus were grown in perlite nourished with Steiner's nutrient solution (Steiner, 1984) under a 14 h/10 h light/dark regime (25°C and 150 µE·m^−2^·s^−1^/22°C) and 30–50% relative humidity in a cultivation room. In all assays, chemical treatment was applied to 10 days old plants. Treatment was infiltrated into the abaxial side of cotyledons using a 1 ml plastic needleless syringe. At least six plants were used for each sample.

*Arabidopsis thaliana* Col-0 and NahG transgenic plants (Delaney et al., 1994) were grown in soil. Surface-sterilized seeds were sown in Jiffy 7 peat pellets and the plants cultivated under a short-day photoperiod (10 h/14 h light/dark regime) at 100–130 µE·m^−2^·s^−1^, 22°C and 70% relative humidity. They were watered with fertilizer-free distilled water as necessary. Plants 4 weeks old were used for all assays. Treatment was applied to three fully developed leaves from one plant, using a 1 ml needless syringe. At least six plants were used for each sample.

Except from concentration dependent assays, aescin was used at 25 µg·ml^−1^ and 10 µg·ml^−1^ concentrations for *B. napus* and *A. thaliana*, respectively. Treatment at these concentrations caused no evident leaf chlorosis symptoms. As a control treatment, EtOH at a corresponding concentration was used.

### Plant Resistance Assays

For *B. napus*- *L. maculans* resistance assays, cotyledons were pre-treated with diverse treatments 4 days prior to infection. Upon infection, the pre-treated cotyledons of *B. napus* plants 14 days old were infiltrated by an aqueous conidial suspension of *L. maculans* JN2-GFP (10^5^ conidia·ml^−1^) as described by Šašek et al. (2012a) using a 1 ml needleless syringe. Prior to inoculation true leaves were removed from plants to avoid cotyledon senescence. At least 12 plants were used per condition. The cotyledons were assessed 11 days after inoculation. The cotyledon areas and lesion areas therein were measured by image analysis using APS Assess 2.0 software (American Phytopathological Society, St. Paul, MN, USA). The relative lesion area was then calculated as the ratio of lesion area to whole leaf area. The hyphal colonization of cotyledons was assessed under a Leica DM5000 B fluorescence microscope (Leica, Germany).

For *A. thaliana* – *P. syringae* pv. tomato DC3000 resistance assays, leaves were pre-treated with aescin 24h prior to infection. The bacteria *Pst* DC3000 was cultivated overnight on lysogeny broth (LB) agar plates with rifampicin at 26°C, then resuspended in 10 mM MgCl_2_ to an OD_600_ of 0.005. The bacterial suspension was infiltrated into three fully developed pre-treated leaves from one plant, using a 1 ml needless syringe. After 3 days, cut leaf discs (one disc per leaf, 0.6 cm in diameter) were collected from infected tissue, with three leaves from a single plant representing one sample. To determine bacterial content in leaves at 0 dpi, samples were collected 1 h after bacterial infiltration. Tissue was homogenized in tubes with silica beads using a FastPrep-24 instrument (MP Biomedicals, Santa Ana, CA, USA). The resulting homogenate was serially diluted and transferred onto LB agar plates with rifampicin. Grown bacterial colonies were counted after 24 h of incubation at 26°C.

### Reactive Oxygen Species Detection

Treated cotyledons were detached and infiltrated under vacuum with diaminobenzidine tetrahydrochloride (DAB; Šašek et al., 2012b) aqueous solution (10 mg·ml^−1^, Sigma–Aldrich), with DAB being solubilized in dimethylformamide. Cotyledons were kept in humid conditions in darkness at room temperature until reddish-brown staining appeared. Chlorophyll was removed using 96% EtOH, after which cotyledons were rehydrated and scanned.

### Analysis of Plant Hormones

Levels of plant hormones were determined 24 hours post treatment in *B. napus* cotyledons. In each sample, 150 mg of fresh material from plant tissue was pooled from eight different plants, as previously described (Dobrev and Kaminek, 2002). Briefly, samples were homogenized with extraction reagent methanol/H_2_O/formic acid (15:4:1, v:v:v) supplemented with stable isotope-labeled internal standards, each at 10 pmol per sample. Clarified supernatants were subjected to solid-phase extraction using Oasis MCX cartridges (Waters Co., Milford, MA, USA), eluates were evaporated to dryness, and the generated solids were dissolved in 30 µL of 15% (v/v) acetonitrile in water. Quantification was done on an Ultimate 3000 high-performance liquid chromatograph (HPLC; Dionex, Bannockburn, IL, USA) coupled to a 3200 Q TRAP hybrid triple quadrupole/linear ion trap mass spectrometer (MS; Applied Biosystems, Foster City, CA, USA), as described by Djilianov et al. (2013). Metabolite levels were expressed in pmol·g^−1^ fresh weight.

### Gene Expression Analysis

Samples (both plant and fungi) were collected 24 hours post treatment. At least six plants were used for each sample for gene expression. Total RNA was isolated from 100 mg of frozen plant tissue or fungal mycelium using a Spectrum Plant Total RNA Kit (Sigma–Aldrich, St. Louis, MO, USA). Next, 1 µg of RNA was treated with a DNA-free Kit (Ambion, Austin, TX, USA) and reverse transcribe to cDNA with M-MLV RNase H Minus Point Mutant reverse transcriptase (Promega Corp., Fitchburg, WI, USA) and anchored oligo dT_21_ primer (Metabion, Martinsried, Germany). Gene transcription was quantified by q-PCR using LightCycler 480 SYBR Green I Master kit and LightCycler 480 (Roche, Basel, Switzerland). The PCR conditions were: 95°C for 10 minutes, followed by 45 cycles of 95°C for 10 s, 55°C for 20 s, and 72°C for 20 s, followed by a melting curve analysis. Relative transcription was calculated with efficiency correction and normalization to the corresponding housekeeping gene for each organism. LmERG3 (Q8J207) and LmERG11 (Q8J1Y7) proteins were retrieved from the Uniprot database and primers were designed for the corresponding genes using PerlPrimer v1.1.21 (Marshall, 2004). Primers are listed in **Supplementary Table 1**.

### Chemical Treatments

Saponins aescin (E1378), hederagenin (H3916), and soyasaponin I (S9951), and fungicides metconazole, fluconazole, boscalid, and fluopyram (all purchased from Sigma–Aldrich, St. Louis, MO, USA) were dissolved in 99.8% ethanol (EtOH) as 10 mg·ml^−1^ stock solution. Tebuconazole, in the form of the commercially formulated product Horizon 250 EW (Bayer CropScience, Germany), was also prepared as 10 mg·ml^−1^ stock solution in EtOH. Ergosterol (Sigma, St. Louis, MO, USA) was prepared as 5 mM stock solution in EtOH and used at the final concentration of 25 µg·ml^−1^. Benzothiadiazole (BTH) was used in the form of the commercially formulated product Bion 50 WG (Syngenta, Switzerland) and prepared directly into the working solutions. The commercial peptide flg22 (EZBiolab) was diluted in Milli-Q water and used at the final concentration of 1 µM. All stock solutions were stored at −20°C.

### Statistical Analyses

If not stated otherwise, all experiments were repeated independently three times, with at least three independent samples (from independent biological material, cultivated under the same conditions). Using Statistica 12 software, statistical analyses were performed either by paired two-tailed Student's *t*-test or by analysis of variance in conjunction with Tukey's honestly significant difference multiple mean comparison *post hoc* test (*P* < 0.05).

## Results

### Aescin Has the Highest Antifungal Activity Among Tested Saponins

**Figure 1:**
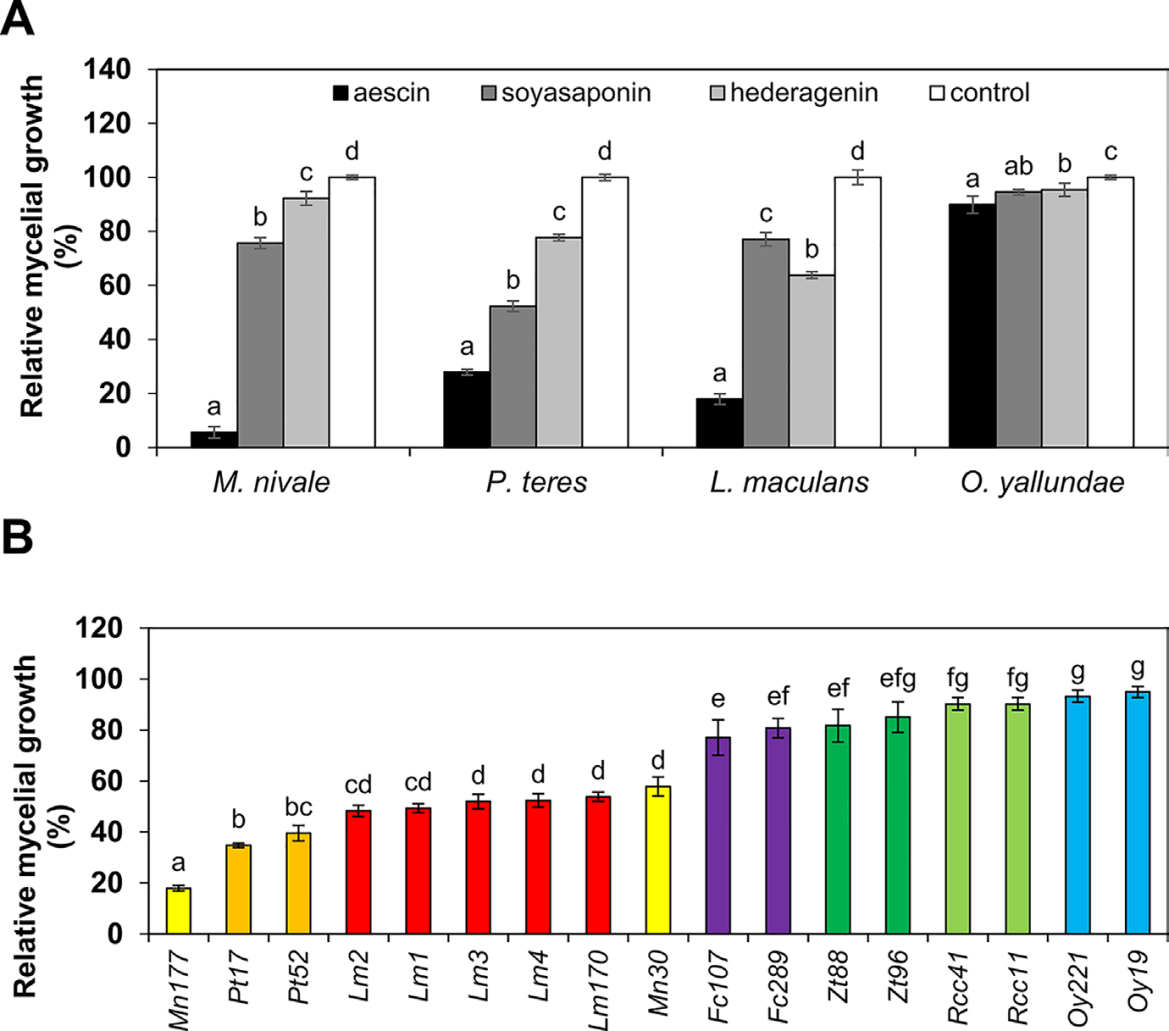
Saponins inhibit mycelial growth of crop pathogens *in vitro* in a species-dependent manner. Relative growth of different fungal species assessed as percentage diameter of fungal colony cultivated on PDA medium supplemented with saponins. The control treatment (without saponins) was set as 100%. **(A)** Growth on aescin (black bars), soyasaponin (dark gray bars), and hederagenin (light gray bars) at 100 µg·ml^−1^, or on a control (without saponin; white bars). The following fungal isolates were used: Mn177, Pt52, Lm1, and Oy19. **(B)** Growth on aescin at the 25 µg·ml^−1^ rate compared to control-treated fungi. All data represent means ± SE from three independent experiments. Different letters above bars illustrate significant differences using ANOVA test in conjunction with Tukey's honestly significant difference multiple mean comparison *post hoc* test (*P* < 0.05). For (A), the statistical analyses were carried out separately within each fungal species (Fc, *Fusarium culmorum*; Lm, *Leptosphaeria maculans*; Mn, *Microdochium nivale*; Oy, *Oculimacula yallundae*; Pt, *Pyrenophora teres*; Rcc, *Ramularia collo-cygni*; Zt, *Zymoseptoria tritici*).

Although saponins are well known to have antifungal activity (Gruiz, 1996; Moses et al., 2014), only very limited data is available quantifying saponin antifungal activity by establishing EC_50_ values. We screened antifungal activity of the triterpenoid plant saponins aescin (from *Aesculus hippocastanum*), soyasaponin (from *Glycine max*), and hederagenin (from *Hedera helix*) against important fungal pathogens (*O. yallundae*, *M. nivale*, *Z. tritici*, *P. teres*, *R. collo-cygni*, *F. culmorum*, and *L. maculans*). These fungi infect such crop plants as wheat, barley, or oilseed rape. Our fungal collection consists of various naturally occurring isolates for each pathogen. To calculate EC_50_ values, we assessed the radial mycelial growth on solid media plates supplemented with saponins. All tested saponins displayed significant antifungal activity, with aescin's activity being the most efficient (**Figure 1A**). Differences in species sensitivity to saponins were observed (**Figure 1A**). As further analyzed for aescin, the activity on isolates varied among species but was mostly conserved within a given fungal species (**Figure 1B**). The fungi most sensitive to saponins were *M. nivale*, *P. teres*, and *L. maculans*, while *O. yallundae*, *R. collo-cygni*, *Z. tritici*, and *F. culmorum* showed only minor growth inhibition (**Figures 1A, B**; **Table 1**). Accordingly, while aescin EC_50_ values for *P. teres*, *M. nivale*, and *L. maculans* isolates occurred in the range of 11–21 µg·ml^−1^, 7–29 µg·ml^−1^, and 25–33 µg·ml^−1^, respectively, EC_50_ values for more-resistant fungal isolates exceeded 100 µg·ml^−1^ and could not be calculated precisely due to concentration limitations caused by saponin solubility (**Table 1**). It is noteworthy that fungal sensitivity (**Figure 1B**) did not correlate with hyphal thickness (**Supplementary Figure 1A**). Correlation between fungal growth rate and fungal sensitivity was observed, however, with the slowly growing isolates being the most resistant (**Supplementary Figure 1B**). In summary, all tested saponins inhibited growth of phytopathogenic fungi in a species-dependent manner, with the strongest growth inhibition provided by aescin.

**Table 1:** Effective dose (EC_50_) values of saponins against pathogenic fungi.

Fungal species	Isolate	EC_50_ [µg·ml^-1^]		
		Aescin	Soyasaponin	Hederagenin
*Microdochium nivale*	Mn30	29.40 ± 6.01	*na*	*na*
	Mn177	6.74 ± 0.84	>100.00	>100.00
*Pyrenophora teres*	Pt17	11.40 ± 9.51	*na*	*na*
	Pt52	20.79 ± 5.14	97.61 ± 8.83	> 100.00
*Leptosphaeria maculans*	Lm170	31.71 ± 3.29	*na*	*na*
	Lm1	28.62 ± 10.03	>100.00	>100.00
	Lm2	25.21 ± 3.25	*na*	*na*
	Lm3	33.11 ± 6.49	*na*	*na*
	Lm4	25.52 ± 0.88	*na*	*na*
*Fusarium culmorum*	Fc107	>100.00	*na*	*na*
	Fc289	>100.00	*na*	*na*
*Zymoseptoria tritici*	Zt88	>100.00	*na*	*na*
	Zt96	>100.00	*na*	*na*
*Ramularia collo cygni*	Rcc11	>100.00	*na*	*na*
	Rcc41	>100.00	*na*	*na*
*Oculimacula yallundae*	Oy19	>100.00	>100.00	>100.00
	Oy221	>100.00	*na*	*na*

EC_50_ values [µg·ml^−1^] calculated by probit analysis for combinations of a given fungal pathogenic isolate and a given saponin, assessed as inhibition of mycelial radial growth on PDA medium with saponin. Data are expressed as means ± SE from three experiments. Cases of EC_50_ > 100.00 indicate that precise values above 100 µg ml^−1^ could not be calculated. na, not analyzed.

### Aescin Antifungal Activity Is Lower Than That of Commercial Fungicides

**Figure 2:**
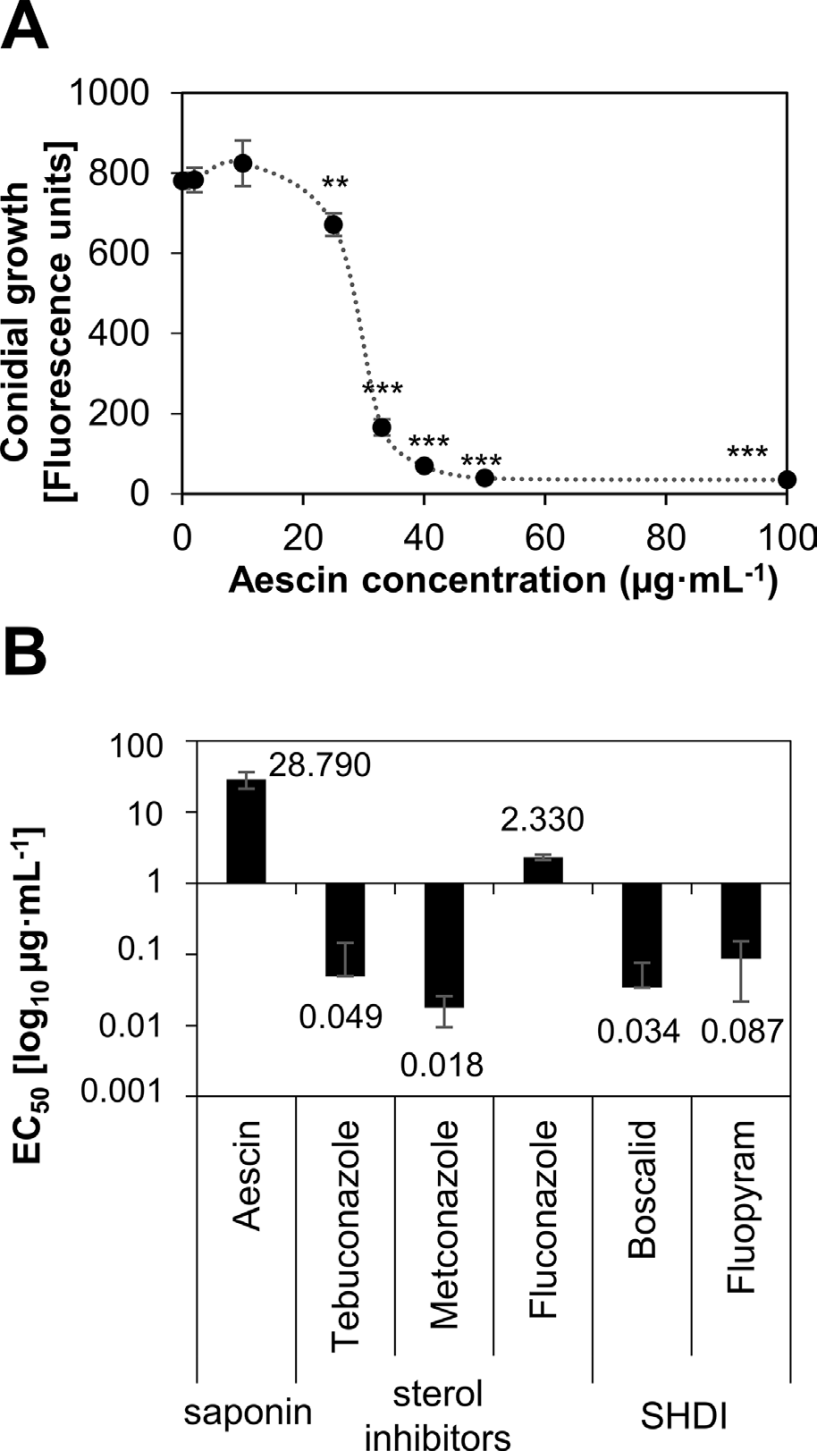
Comparison of aescin and fungicide inhibitory activity against *L. maculans*. Growth of *L. maculans* JN2-GFP conidia *in vitro* in Gamborg liquid medium supplemented with aescin, fungicides, or control medium assessed as GFP fluorescence at 3 days. **(A)** The concentration-dependent curve for growth in the presence of aescin. Data are mean ± SE of absolute fluorescence units out of three experiments. Asterisks indicate significant differences between aescin treatment and control (0) using two-tailed Student's *t*-test (**P < 0.01; ***P < 0.001). **(B)** Calculated EC_50_ values [µg·ml^−1^] ± SE at log_10_ base for aescin and different fungicides from sterol inhibitor (tebuconazole, metconazole, and fluconazole) and succinate dehydrogenase inhibitor (SHDI) classes (boscalid, fluopyram). Data are from three independent experiments.

The biological activity of aescin was further studied on *L. maculans*, which is a destructive pathogen of *B. napus*. The antifungal activities (EC_50_ values) of aescin and synthetic fungicides were first compared. Several fungicides of different classes were tested, including triazolic sterol inhibitor tebuconazole, commonly used for *B. napus* protection against phoma stem canker (Child et al., 1993). For this purpose, fungal growth was measured as GFP fluorescence of germinating conidia of the *L. maculans* JN2 isolate expressing GFP (JN2-GFP) (Balesdent et al., 2001; Šašek et al., 2012a). In this setup, aescin was fully fungitoxic to the conidia at concentrations above 50 µg·ml^−1^ (**Figure 2A**) and demonstrated EC_50_ of 28.79 µg·ml^−1^ (**Figures 2A, B**) that was in agreement with EC_50_ obtained for the *L. maculans* field isolates (**Table 1**). EC_50_ values for the fungicides were mostly in a range from 0.018 µg·ml^−1^ to 0.087 µg·ml^−1^, with metconazole being the most efficient (**Figure 2B**). On the other hand, fluconazole, was the least efficient (EC_50_ = 2.33 µg·ml^−1^; **Figure 2B**). Overall, aescin inhibits conidial and mycelial growth of *L. maculans in vitro* and demonstrates antifungal activity 1 to 3 orders of magnitude lower than that of fungicides.

### Antifungal Activity of Aescin Against *L. Maculans* Occurs Through Its Interaction With Sterols

**Figure 3:**
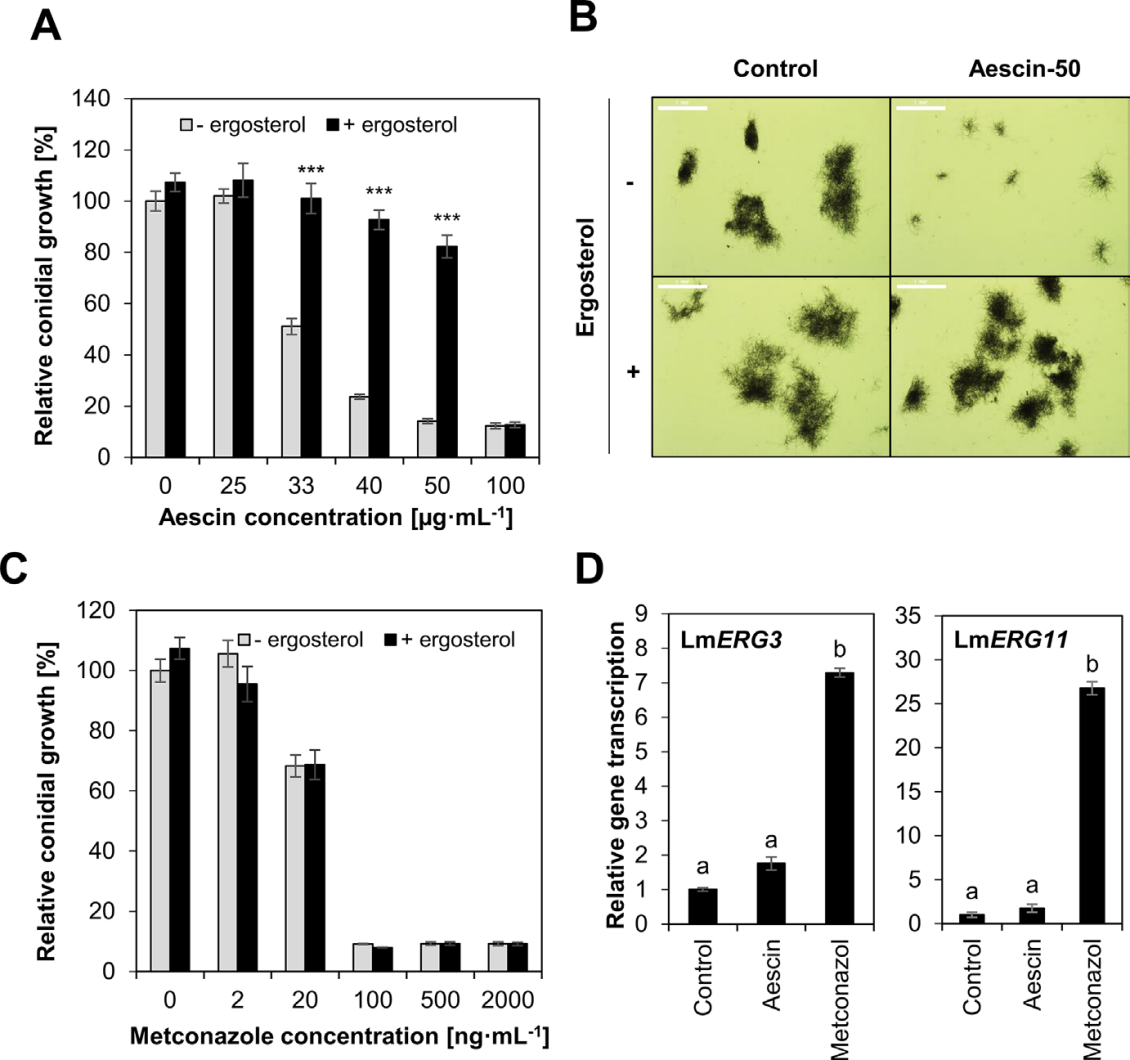
Ergosterol reverts aescin-mediated growth inhibition of *L. maculans*. **(A**–**C)**. Growth of *L. maculans* JN2-GFP conidia *in vitro* in Gamborg liquid medium supplemented with aescin **(A**, **B)** or metconazole **(C)** in absence (gray bars) or presence (black bars) of ergosterol (25 µg·ml^−1^). **(A**, **C)** Data are expressed as relative fluorescence units at 4 days of growth compared to control (0) without ergosterol, set as 100%. Data are expressed as means ± SE from three independent experiments. Asterisks indicate significant differences (***P < 0.001; two-tailed Student's *t*-test) between treatments with and without ergosterol for each concentration of aescin or metconazole. **(B)** Light microscopy of germinating hyphae at control and aescin at 50 µg·ml^−1^ rate at 5 days of growth in presence or absence of ergosterol (25 µg·ml^−1^). Scale bar corresponds to 1 mm. **(D)** Relative transcription of ergosterol biosynthetic genes *LmERG3* and *LmERG11* at mycelium 7 days old and treated with aescin (100 µg·ml^−1^) or metconazole (2 µg·ml^−1^) for 24 h. Gene transcription was analyzed by qPCR, normalized to *LmTubulin*, then compared to control treatment. Data represent mean ± SE from one biological experiment (three biological replicates) representative of three. Different letters above bars illustrate significant differences using ANOVA test in conjunction with Tukey's honestly significant difference multiple mean comparison *post hoc* test (*P* < 0.05).

Aescin's antimicrobial effect occurs through interference with membranes and interaction with sterols (Morrissey and Osbourn, 1999; Sreij et al., 2019). Therefore, we tested aescin's activity in the presence of ergosterol, a sterol naturally present in fungal membranes. Ergosterol markedly restored the growth of *L. maculans* JN2-GFP in the presence of aescin at all the inhibiting concentrations (**Figure 3A**), which was confirmed also by microscopic analysis of hyphae (**Figure 3B**) (**Supplementary Figure 2**). Growth inhibition caused by metconazole could not be reversed by the ergosterol supply (**Figure 3C**). Ergosterol itself did not significantly affect fungal growth (concentration 0 of **Figures 3A, C**). Inasmuch as triazole fungicides block biosynthesis of ergosterol (Sanati et al., 1997), transcription of *LmErg3* and *LmErg11* genes, identified as involved in ergosterol biosynthesis in *L. maculans* (Griffiths and Howlett, 2002), was assessed following aescin treatment of the fungus. The effect of aescin or fungicides was observed in 7-day-old *L. maculans* culture 24 h post treatment. While metconazole induced transcription of *LmErg3* and *LmErg11* genes by 7 times and 27 times, respectively, in excess of the control, aescin did not significantly upregulate transcription of these genes (**Figure 3D**). The data show that aescin interfered with the fungal ergosterol but not directly with its biosynthesis.

### Aescin Pretreatment Confers Resistance in *B. napus* Against *L. maculans*

**Figure 4:**
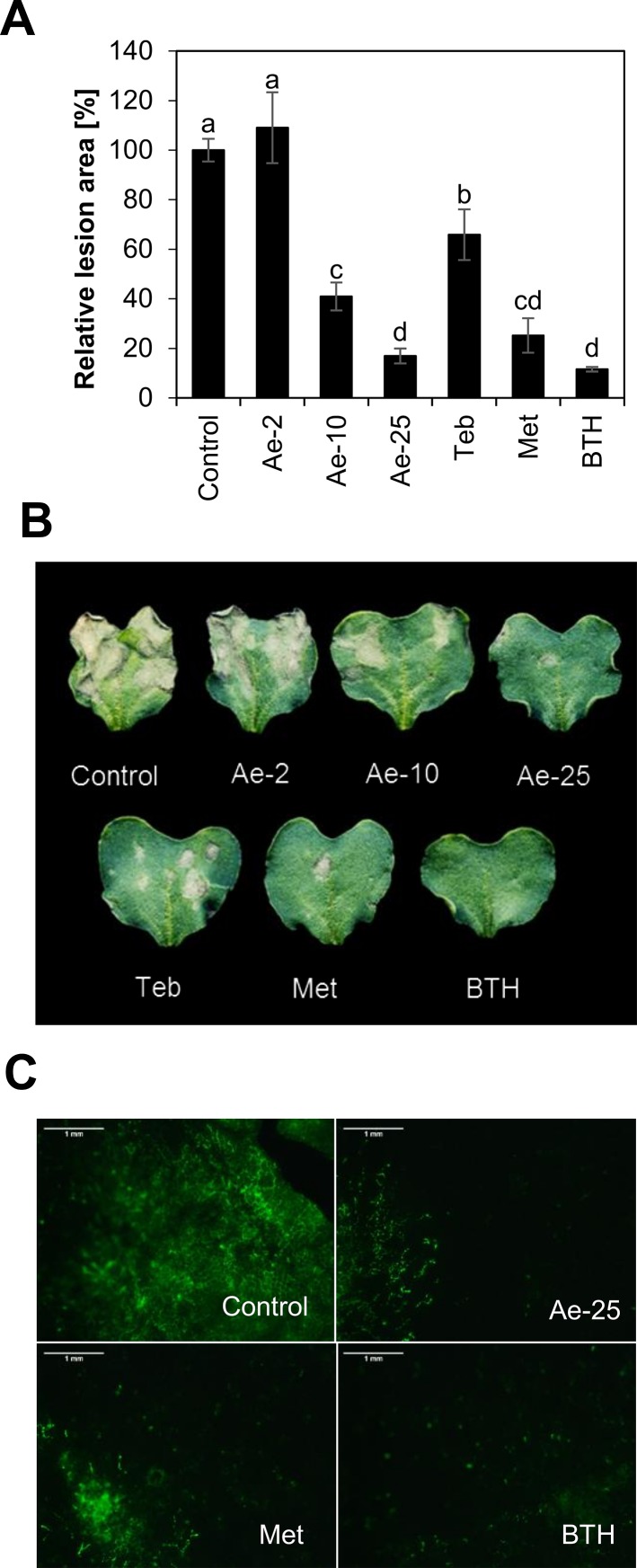
Aescin pretreatment provides B. napus with efficient resistance against *L. maculans*. Cotyledons of *B. napus* were infiltrated by aqueous solutions of aescin (Ae; at 2, 10, and 25 µg·ml^−1^), tebuconazole, metconazole (Teb and Met; both at 2 µg·ml^−1^), BTH (30 µM), or a control 3 days prior to being infiltrated by conidial suspension of *L. maculans* JN2-GFP. The outcome was assessed at 12 days. **(A)** Quantification of the relative lesion area by image analysis is expressed as percentage. Control treatment was set as 100%. Data represent means ± SE from five independent experiments. Different letters above bars illustrate significant differences using ANOVA test in conjunction with Tukey's honestly significant difference multiple mean comparison *post hoc* test (*P* 0.05). **(B)** Panel with representative infected cotyledons. **(C)** Hyphal spread of JN2-GFP fungus in infected cotyledons. Scale bar corresponds to 1 mm.

Given the antifungal activity of aescin, we further investigated whether pretreatment with aescin could efficiently protect *B. napus* against *L. maculans*. Pretreatment of *B. napus* cotyledons by leaf infiltration with aescin at rates of 25 µg·ml^−1^ and 10 µg·ml^−1^ 3 days prior to inoculation with *L. maculans* JN2-GFP efficiently reduced the cotyledon area covered by necrotic lesions (**Figures 4A, B**). The effect was comparable to those provided both by the fungicide metconazole at rate 2 µg·ml^−1^ and the plant defense inducer benzothiadiazole (BTH) at rate 30 µM. BTH activates the *B. napus* immune system and provides protection against *L. maculans* (Šašek et al., 2012a). The protection provided by aescin was even more efficient than was that induced by the fungicide tebuconazole at rate 2 µg·ml^−1^. Aescin's protection was concentration dependent, and no significant effect was observed with aescin at the 2 µg·ml^−1^ level. Microscopic analyses (**Figure 4C**) revealed only a few restricted GFP-fluorescent hyphal zones in aescin- and metconazole-pretreated cotyledons, while the control treatment displayed extensive hyphal network all over the infected cotyledon and corresponding to the localization of necroses. We also showed that foliar spray of aescin aqueous solution is protective (**Supplementary Figure 3**), although higher concentration may be required compared to when infiltration is used. Taken together, our data demonstrate that aescin protects *B. napus* against *L. maculans* by inhibiting tissue colonization by fungal hyphae and necrosis formation. It is noteworthy that the treatment with aescin at concentration 25 µg·ml^−1^ decreased cotyledon growth to a similar extent as did 30 µM BTH (**Supplementary Figure 4**). At higher concentrations (above 50 µg·ml^−1^), aescin caused chlorosis and necroses on leaves (**Supplementary Figure 4**). Treatment with 10 µg·ml^−1^ of aescin caused no obvious effects on cotyledon fitness (**Supplementary Figure 4**), however, and this concentration was still able to reduce *L. maculans* infection (**Figure 4A**).

### Aescin Induces Defense Responses in *L. Maculans*, Governed by SA Pathway and Oxidative Burst

**Figure 5:**
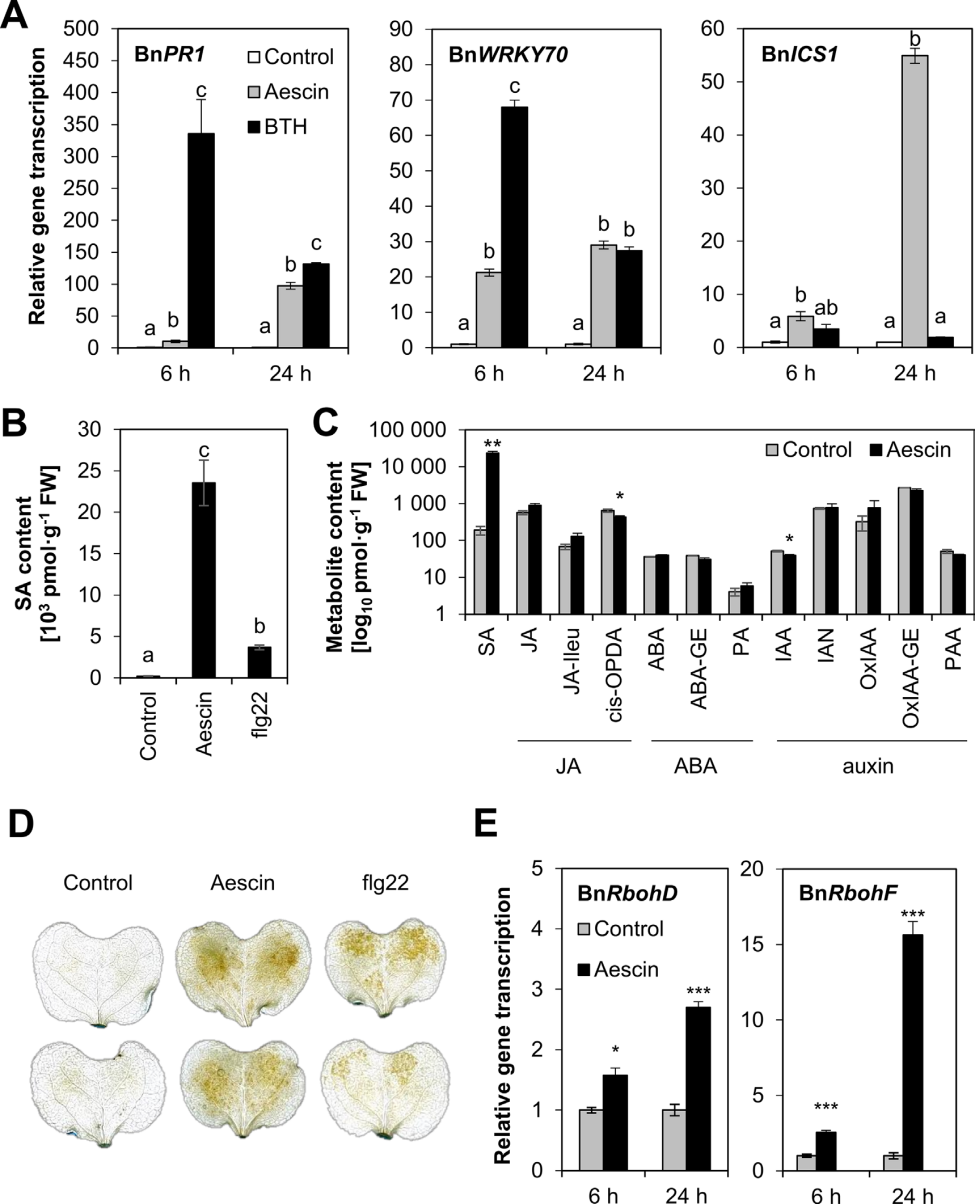
Aescin treatment triggers defense responses in *B. napus*. Cotyledons of *B. napus* were infiltrated by aqueous solutions of aescin (25 µg·ml^−1^), BTH (30 µM), flg22 (1 µM), or a control treatment. **(A)** Gene transcription of *pathogenesis-related BnPR1*, *BnWRKY70*, and *isochorismate-synthase 1 BnICS1* was analyzed by qPCR after 6 and 24 h of treatment, normalized to *BnActin* and *BnTIP41*, and compared to the corresponding control at 6 or 24 h (set as 1). Data represent mean ± SE from one biological experiment (four biological replicates), representative of three. **(B**, **C)** Content of salicylic acid (SA; **B**) and SA, JA, ABA- and auxin-derived hormones in control- or aescin-treated plants **(C)**. The content of hormones in plant tissue expressed as pmol·g^−1^ fresh weight ± SE was measured after 24 h. Data are means of four biological replicates. Experiment was repeated twice. SA, salicylic acid; JA, jasmonic acid; JA-Ile, JA-isoleucine; *cis*-OPDA, *cis*-12-oxo-phytodienoic acid; ABA, abscisic acid; ABA-GE, ABA-glucose ester; PA, phaseic acid; IAA, indole-3-acetic acid; OxIAA, oxo-IAA; OxIAA-GE, oxo-IAA-glucose ester; IAN, indole-3-acetonitrile; PAA, phenylacetic acid. **(D)** Oxidative burst visualized by DAB staining at 24 h post treatment. Images are representative of three experiments. **(E)** Transcription of respiratory burst oxidase homolog *RbohD* and *RbohF* genes following aescin treatment was analyzed at 6 or 24 h by qPCR, normalized to Bn*Actin* and Bn*TIP41*, and compared to the corresponding control (set as 1). Data represent means ± SE from one biological experiment (four biological replicates) representative of three. For **(A)** and **(B)**, different letters above bars illustrate significant differences using ANOVA test in conjunction with Tukey's honestly significant difference multiple mean comparison *post hoc* test (*P* 0.05). For **(A)**, the statistical analyses were carried out separately within each time point. For **(C)** and **(E)**, asterisks indicate significant differences between control and a given treatment (*P < 0.05; **P < 0.01; ***P < 0.001; two-tailed Student's *t*-test).

The fact that aescin can provide a higher level of plant protection than do fungicides having more potent antifungal activity suggested a possibility that aescin stimulates plant defense. Therefore, transcription of plant defense marker genes was determined in cotyledons 6 h and 24 h after treatment with aescin or BTH (**Figure 5A**). At both time points, aescin upregulated transcription of *BnPR1* and SA-specific transcription factor *BnWRKY70* genes previously characterized as being marker genes of activated SA pathway in *B. napus* (Šašek et al., 2012b). At 24 h, the level of induction was similar to that of BTH, but aescin and BTH induced defense genes with different kinetics. In contrast to BTH, aescin also upregulated transcription of the SA-biosynthetic gene for isochorismate synthase 1 (*BnICS1*). Given the strong induction of *BnICS1* transcription, aescin's capacity to stimulate SA production was tested and compared to that of flg22, a well-characterized microbe-associated molecular pattern (MAMP) activating SA pathway in *A. thaliana* (Tsuda et al., 2008; Lloyd et al., 2014). Aescin application at the 25 µg·ml^−1^ rate to cotyledons led to a massive increase in SA 24 h after treatment, with SA content reaching even higher levels than those seen following treatment with 1 µM flg22 (**Figure 5B**). Other tested phytohormone metabolites were altered not at all or only slightly by aescin (**Figure 5C**). Aescin caused mild decrease in the *cis*-OPDA metabolite, the JA precursor (Dave and Graham, 2012), and auxin forms. In summary, based on gene transcription analysis and phytohormone measurement, it was apparent that aescin treatment activated the SA pathway.

Further defense responses were analyzed in aescin-treated *B. napus* cotyledons. At 24 h following treatment aescin triggered accumulation of ROS compared to the control treatment, as was visualized by brown-reddish precipitates in DAB staining assay (**Figure 5D**). The accumulation was induced to a similar extent as was that for the flg22 treatment and was concentration dependent (**Supplementary Figure 5**). Accordingly, at 24 h post treatment, aescin induced transcription of respiratory burst oxidase homolog *RbohD* and *RbohF* genes coding NADPH oxidases responsible for ROS production in plants after exposure to MAMPs (Torres et al., 2005; Qi et al., 2017) (**Figure 5E**). The fungicides tebuconazole and metconazole did not elicit transcription of any defense genes, nor did they trigger oxidative burst in *B. napus* cotyledons (**Supplementary Figures 6A, B**).

### Aescin-Induced SA-Dependent Resistance to Bacterial Pathogen in *A. thaliana*

**Figure 6:**
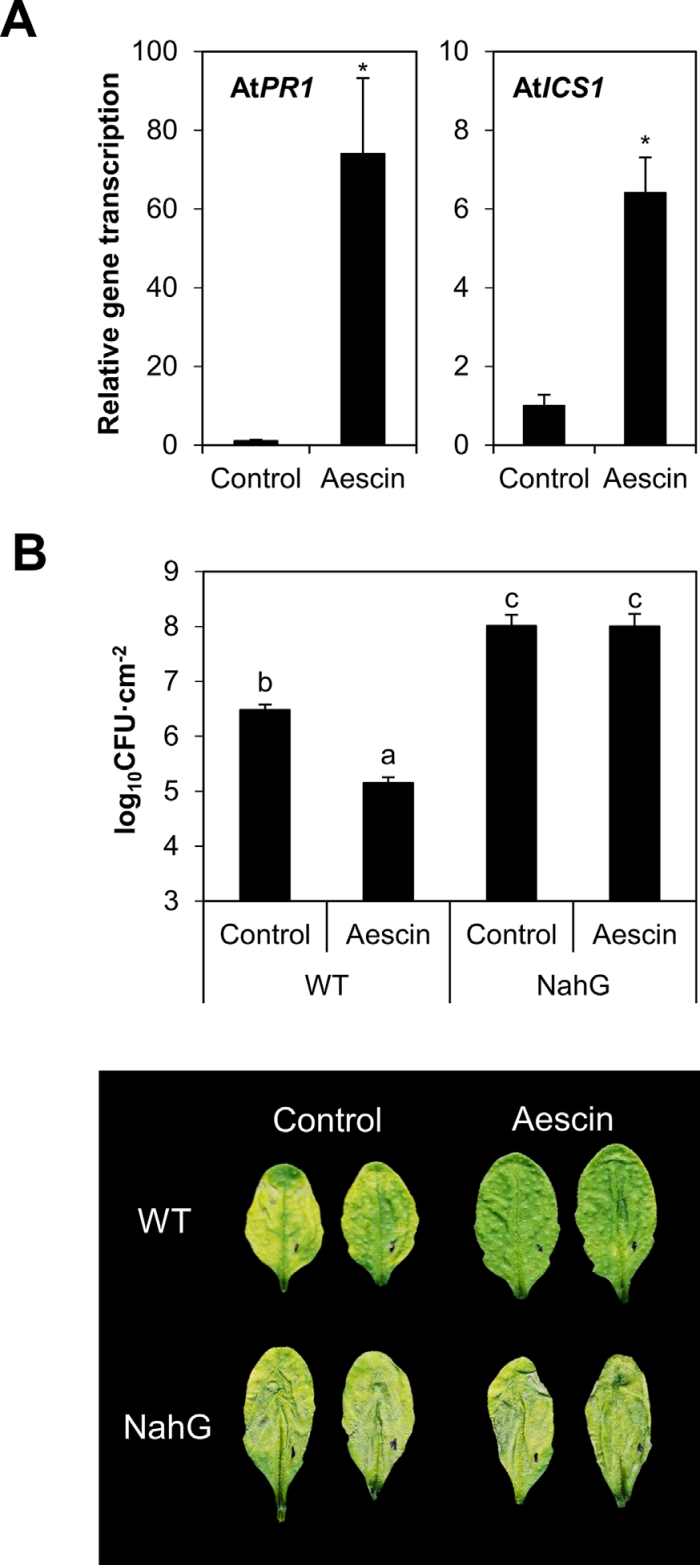
Aescin pretreatment triggers defense gene transcription in *A. thaliana* and resistance against bacteria *Pseudomonas syringae* pv. tomato DC3000 (Pst DC3000). Leaves of Arabidopsis plants were infiltrated by aqueous solutions of aescin (10 µg·ml^−1^) or a control treatment. **(A)** Gene transcription of *pathogenesis-related AtPR1* and *isochorismate-synthase 1 AtICS1* was analyzed by qPCR after 24 h of treatment, normalized to At*TIP41*, then compared to the control. Data represent mean ± SE from one biological experiment (four biological replicates), representative of three. Asterisks indicate significant differences *P < 0.05; two-tailed Student's *t*-test). **(B)** Bacterial growth of *Pst* DC3000 bacteria at 3 days post inoculation in infected leaves of wild type (WT) or NahG transgenic plants pretreated by aescin or control for 24 h. (*upper*) Bacterial titers within leaves. Data represent means of colony forming units (CFU) per cm^2^ ± SE from six independent replicates of one experiment, representative of three. Different letters above bars illustrate statistical difference between samples using ANOVA with a Tukey honestly significant difference multiple mean comparison *post hoc* test (*P* < 0.01). (*lower*) A representative leaf for each treatment is shown.

To exclude that the phenomenon of aescin-activated immunity is specific to the *B. napus–L. maculans* system, the activity of aescin was investigated also in an *A. thaliana* model system challenged by a hemibiotrophic bacterial pathogen, *Pst* DC3000. After 24 h of treatment with aescin at the 10 µg·ml^−1^ level, there was upregulated transcription of *AtPR1* and *AtICS1* genes in *A. thaliana* leaves (**Figure 6A**). Aescin pretreatment for 24 h also led to induced resistance against bacterium *Pst* DC3000, observed as substantial decrease of both disease symptoms and bacterial titers in infected leaves (**Figure 6B**). For direct investigation of possible SA involvement in aescin-triggered resistance to Pst DC3000, we used NahG transgenic plants, in which low endogenous SA levels are maintained through the expression of SA-hydroxylase (Delaney et al., 1994). In NahG plants, the effect of aescin-induced resistance against *Pst* DC3000 was lost (**Figure 6B**).

Aescin did not impact the growth of *Pst* DC3000 cultivated *in vitro* (**Supplementary Figure 7A**). It also did not affect the bacterial titers in aescin-pretreated leaves sampled 1 h after infection with *Pst* DC3000 (**Supplementary Figure 7B**). In addition, co-inoculation of *A. thaliana* plants simultaneously with *Pst* DC3000 bacterium and aescin did not affect the bacterial colonization in the infected leaves (**Supplementary Figure 7C**). These data suggest that the bacterial resistance provided by aescin in *A. thaliana* is not due to a direct antibacterial effect. Together, these data show increased resistance of *A. thaliana* against *Pst* DC3000 induced by aescin treatment, which possibly acts through activating SA-dependent immune pathways.

## Discussion

Currently, field crops are protected from fungal pathogens by such fungicide compounds as benzimidazoles, sterol biosynthesis inhibitors, strobilurins, or succinate dehydrogenase inhibitors. Because the occurrence of synthetic pesticide residues is progressively degrading the health of living organisms and the environment even as fungicide resistance is developing, there is a clear need to discover "greener" antifungal agents. Our study was focused on plant-derived saponins as hypothetical new plant protectants.

### Aescin: A Potent Antifungal Saponin

The effect of saponins on fungi has been widely studied (Gruiz, 1996; Barile et al., 2007; Hoagland, 2009; Saha et al., 2010; Teshima et al., 2013). Heretofore, however, there has been only few comprehensive studies of saponin activity against phytopathogens, including to determine EC_50_ values and compare more deeply saponin efficiency with that of synthetic fungicides.

EC_50_ values in the ranges 181–678 µg·ml^−1^ and 230–455 µg·ml^−1^ have been reported for the inhibitory activity of saponins of *Sapindus mukorossi* and *Diploknema butyracea*, respectively, on mycelial growth of phytopathogens *Rhizoctonia* sp. and *Sclerotinia* sp. (Saha et al., 2010). Minutosides extracted from *A. minutiﬂorum* have been shown to be highly inhibitory to spore germination of soil- and air-borne fungi (*Fusarium oxysporum*, *F. solani*, *Pythium ultimum*, *Rhizoctonia solani*, *Botrytis cinerea*, *Alternaria alternata*, *A. porri*, and *Trichoderma harzianum*) at 10–1000 µg·ml^−1^, depending upon the individual fungal species and saponin (Barile et al., 2007). The antifungal activity of aescin, a saponin from horse chestnut *Aesculus hippocastanum*, has been characterized only poorly. Previous studies have reported both antibacterial activity of β-aescin towards soil *Rhizobium* bacteria (Zablotowicz et al., 1996) and its antifungal activity against Candida sp. (Franiczek et al., 2015). However, knowledge as to aescin's activity against phytopathogens has not previously been presented. Here, we tested the antifungal effect of aescin on seven species of phytopathogenic fungi causing crop losses in cereals and rapeseed. The activity was also tested in comparison to that of soyasaponin, hederagenin, and synthetic fungicides.

We have shown here that aescin displayed strong inhibitory effect against fungal growth, significantly impeding mycelial growth in all tested fungal isolates (**Figure 1A**). Aescin was highly active against *M. nivale*, *P. teres*, and *L. maculans*, exhibiting EC_50_ values below 50 µg·ml^−1^ (**Table 1**). Aescin also exhibited greater antifungal activity than did the other two saponins tested, soyasaponin and hederagenin (**Figure 1A**, **Table 1**). In light of these results and those of previous studies on other saponins, aescin emerges as a potent antifungal saponin. A parallel comparison of aescin's inhibitory activity with those of synthetic commercial fungicides was carried out on germinating *L. maculans* conidia. Aescin's EC_50_ was from 1 to 3 orders of magnitude greater in comparison to that of fungicides (**Figure 2**). Co-treatment with ergosterol, which reverses the effect of aescin but not the effect of fungicides, showed aescin to have a different mode of action on membranes compared to that of fungicides (**Figures 3A, C**).

We observed aescin activity to be variable in different fungal species, while it was mostly conserved among isolates within a given species (**Figure 1B**). Compared to other fungi, O. *yallundae* isolates were the most resistant to aescin and the other tested saponins (**Figure 1**, **Table 1**). This general resistance of *O. yallundae* independent of saponin type (**Figure 1A**) may reflect its different fungal morphology and physiology. A correlation was observed between growth rate and fungal sensitivity, and *O. yallundae* is a slowly growing fungus (**Supplementary Figure 1**). Furthermore, saponin-resistant fungi may contain membranes with low sterol content (Arneson and Durbin, 1967; Barile et al., 2007) or fungal sterols with moieties bound only weakly by saponins (Steel and Drysdale, 1988). In general, fungi with defective sterol biosynthesis or in the presence of sterol inhibitors are more resistant to saponins (Olsen, 1973; Defago and Kern, 1983). Moreover, some fungi can cleave sugar moieties of saponins, thereby resulting in non-toxic molecules. For some saponins, a C3-attached sugar moiety or moieties can be critical for both permeabilizing membrane and antifungal properties of saponins (Morrissey and Osbourn, 1999). For instance, *Gaeumannomyces graminis* and *Gibberella pulicaris* produce avenacinase and alpha-chaconinase, respectively, and these detoxify their hosts' saponins (Bowyer et al., 1995; Becker and Weltring, 1998). To sum up, our study characterizes the fungistatic activity of aescin on different phytopathogenic fungi and provides a parallel comparison to fungicides.

### Aescin: A Potent Plant Disease Control Agent

The role of saponins as plant-protecting compounds has been shown. Namely, avenacin triterpene glycosides protect oat roots against soil-borne fungal pathogens such as the *Gaeumannomyces graminis* causing disease "take all" in cereals (Papadopoulou et al., 1999). Saponin alliospiroside extracted from *A. cepa* protects strawberry plants against *C. gloeosporioides*, the causal agent of anthracnose (Teshima et al., 2013). Beta-amyrin-derived triterpene glycosides confer resistance in *Barbarea vulgaris* against flea beetle (*Phyllotreta nemorum*) (Nielsen et al., 2010). Here, we showed that pretreatment of *B. napus* cotyledons with aescin led to strong concentration-dependent plant protection against infection by the hemibiotrophic fungus *L. maculans* that causes phoma stem canker. This was demonstrated also by the reduced hyphal spread and necrosis formation in infected cotyledons pretreated with aescin (**Figure 4**).

Aescin induced transcription of SA-dependent genes in *B. napus*. Namely, aescin led to increased transcription of the SA biosynthetic gene *BnICS1* (**Figure 5A**) and caused great accumulation of SA (**Figure 5B**). Additionally, aescin triggered oxidative burst, as demonstrated by ROS accumulation and upregulated transcription of *BnRbohD* and *BnRbohF* genes (**Figure 5E**). Both SA and oxidative stress have antimicrobial properties (Lamb and Dixon, 1997). Aescin's dual mode of action combining antifungal and induced plant immune responses led to a very efficient inhibition of blackleg disease on *B. napus*. Aescin treatment provided plant resistance to a similar extent as did the fungicide metconazole or BTH (**Figure 4A**), a potent plant immunity inducer (Zhou and Wang, 2018). The key role played by triggering immunity in aescin-induced *B. napus* protection is seen in the fact that metconazole is greater than 1000 times more effective in its antifungal activity against *L. maculans* compared to aescin (**Figures 2B** and **3A, C**). Overall, then, the plant defense activation may be an important part – and perhaps the crucial part – of aescin-induced plant protection. In the animal kingdom, various studies have shown that saponins induce immunity in vertebrates. Indeed, they are commonly used as vaccine adjuvants (Sun et al., 2009; Moses et al., 2014) because they stimulate antibody production (Soltysik et al., 1995), production of cytotoxic T-lymphocytes or induce inflammasome (Marty-Roix et al., 2016). To the best of our knowledge, we are the first to show that saponins may induce plant immune responses.

### SA Pathway: Target of Aescin-Triggered Immunity

Our data show that aescin activates the plant immune system, and specifically the SA pathway, in both *B. napus* and *A. thaliana*. The SA pathway was shown to be the main defense route activated in *B. napus* upon *L. maculans* infection (Potlakayala et al., 2007; Šašek et al., 2012b). Various microorganisms evolved strategies to disrupt SA-mediated defense (Qi et al., 2018). Some *L. maculans* effectors, such as AvrLm4-7, may target this pathway to weaken the host immune system (Nováková et al., 2016). *B. napus* plants transformed with the salicylate hydroxylase gene *nahG* have been shown to have compromised systemic acquired resistance against *L. maculans* and *P. syringae* pv. *maculicola* (Potlakayala et al., 2007). In comparison with SA, other tested phytohormone metabolites were not or much less affected in *B. napus*. Slight decrease in *cis*-OPDA metabolite might be caused by SA-mediated repression on JA pathways, as has been described for *A. thaliana* (Pieterse et al., 2009; Dave and Graham, 2012).

The crucial role of SA in aescin-triggered plant resistance against pathogens was shown using the *A. thaliana*–*P. syringae* model pathosystem (Katagiri et al., 2002). Leaf pretreatment with aescin strongly inhibited *Pst* DC3000 infection (**Figure 6B**). The protective effect of aescin relied on the active defense mechanisms of *A. thaliana* inasmuch as aescin did not exhibit direct antibacterial properties (**Supplementary Figure 7B**). Accordingly, the treatment with aescin simultaneously with the infection had no effect on *Pst* DC3000 infection (**Supplementary Figure 7A**), thus suggesting some time is required to activate the plant defense. Furthermore, NahG plants defective in SA pathway showed no effect of aescin on the bacterial infection (**Figure 6B**), thus demonstrating that a functional SA pathway is indispensable for aescin-induced *A. thaliana* resistance against *Pst* DC3000.

In conclusion, we report here broad-spectrum antifungal activity of aescin and the new finding that aescin elicits defense responses in *B. napus* and *A. thaliana* by triggering the SA pathway and oxidative burst. These responses lead ultimately to highly efficient protection of *B. napus* against the fungus *L. maculans* and of *A. thaliana* against the bacteria *Pst* DC3000. The effect of aescin against *L. maculans* is of an extent comparable to that provided by fungicide protection. Additionally, we showed that aescin provides protective activity as a foliar spray. Taken together, our results suggest that aescin may constitute an attractive bioactive molecule with dual mode of action that could be found suitable for field application.

## Data Availability Statement

The datasets generated for this study are available on request to the corresponding author.

## Author Contributions

LT and PM designed the experiments. LT, MJ, DM, RP, PD, and PM performed the experiments. LT, MJ, PD and PM analyzed the data. LT, MJ, and PM wrote the manuscript. LB revised the manuscript and provided a methodological and knowledge platform for studying the *L. maculans* and *B. napus* interaction, finances, and lab space for a substantial part of the work. All the authors discussed the results and commented on the manuscript.

## Funding

The research leading to these results was supported by projects QJ1310226, QK1910197, MZE-RO1118, TA ČR GAMA PP1 TG03010009, by the Ministry of Education, Youth and Sports of the Czech Republic from the European Regional Development Fund-Project 'Centre for Experimental Plant Biology': No. CZ.02. 1.01/0.0/0.0/16_019/0000738, and the European Structural and Investment Funds, OP RDE-funded project 'CHEMFELLS4UCTP' (No. CZ.02.2.69/0.0/0.0/17_050/0008485).

## Conflict of Interest

Author Pavel Matusinsky is employed by company Agrotest Fyto, Ltd. The remaining authors declare that the research was conducted in the absence of any commercial or financial relationships that could be construed as a potential conflict of interest.
