# Supplementary material for: Dual Mode of the Saponin Aescin in Plant Protection: Antifungal Agent and Plant Defense Elicitor
Source: Front Plant Sci. 2019 Nov 28;10:1448. doi: 10.3389/fpls.2019.01448 (PMC6893899; doi:10.3389/fpls.2019.01448)
Supplement: Supplementary file 1 [file DataSheet_1.pdf]

Supplementary Figure 1

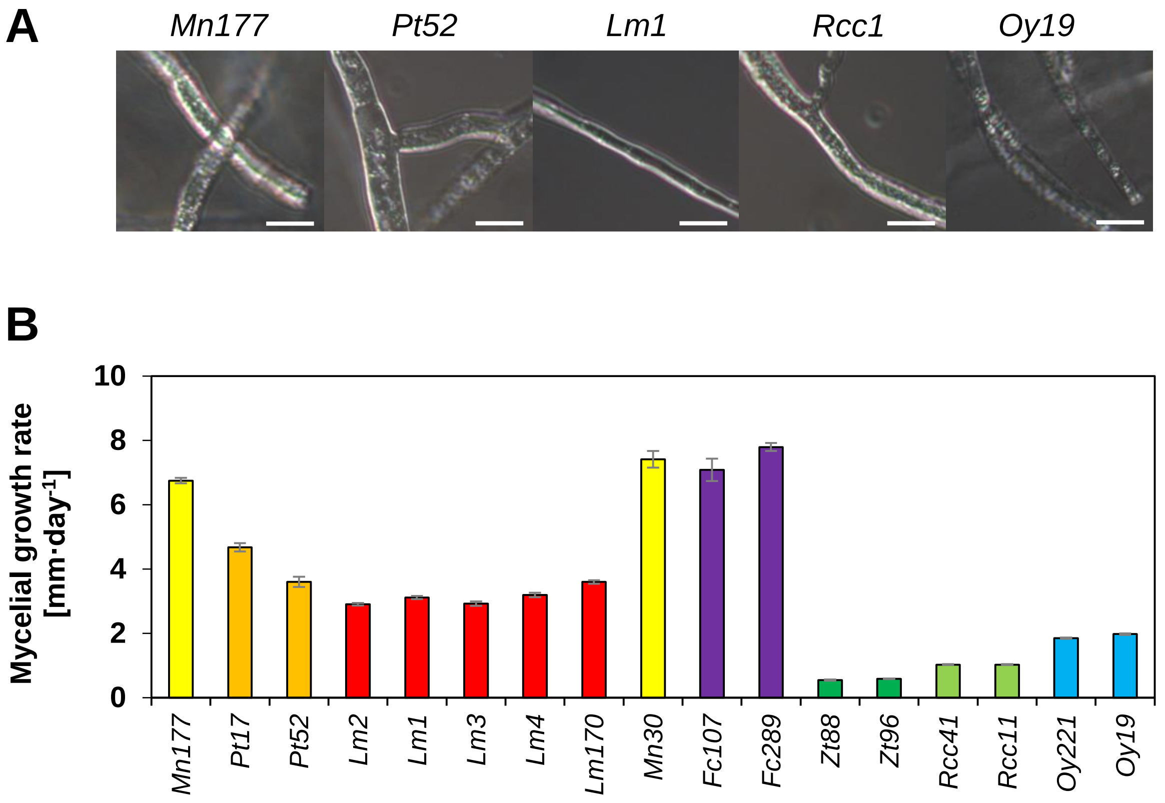

Supplementary Figure 1 | Hyphal morphology and growth rate across different fungal species and isolates. (A) Hyphal segments from outer edge of fungal colonies grown on PDA plate for 10 days. Scale bar corresponds to 10 µm. (**B)** Growth rate of fungal colony expressed as mm grown per day. The data represent means ± SE from three independent experiments.

Supplementary Figure 2

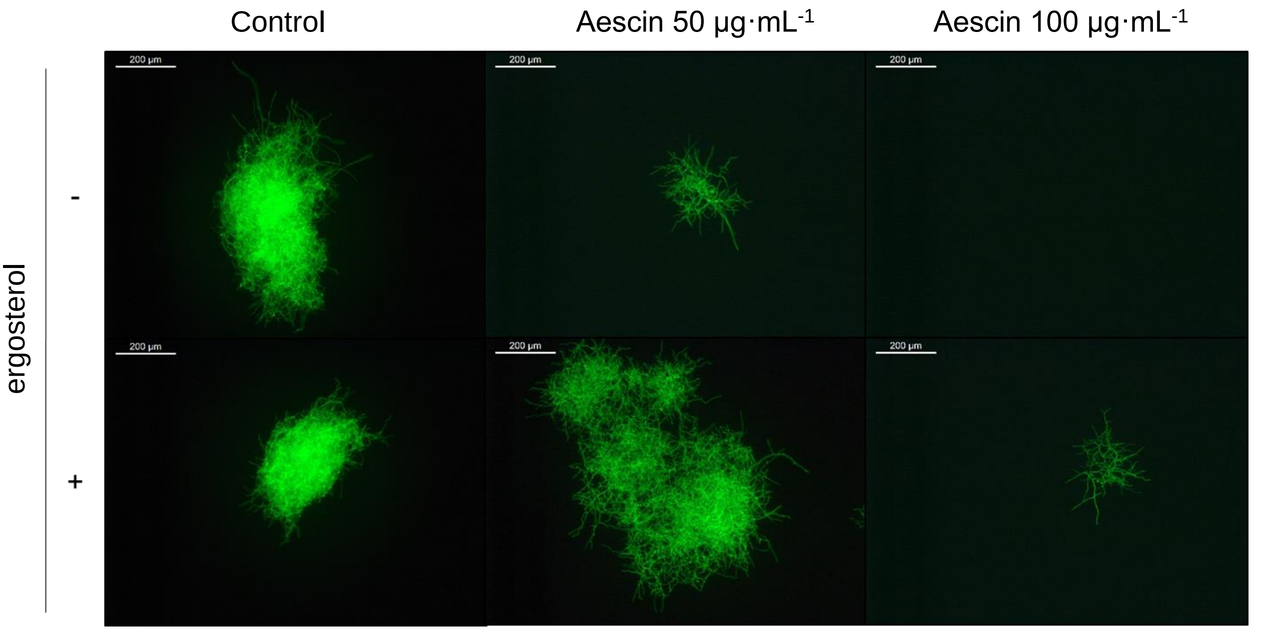

Supplementary Figure 2 | Ergosterol at least partly decreases block in *L. maculans* growth caused by aescin at 100 µg·mL^{-1} rate. Growth of *L. maculans* JN2-GFP conidia *in vitro* in Gamborg liquid medium supplemented with aescin (50 or 100 µg·mL^{-1}) with or without added ergosterol (25 µg·mL^{-1}) assessed by epifluorescence microscopy at 5 days. Scale bar corresponds to 200 µm.

Supplementary Figure 3

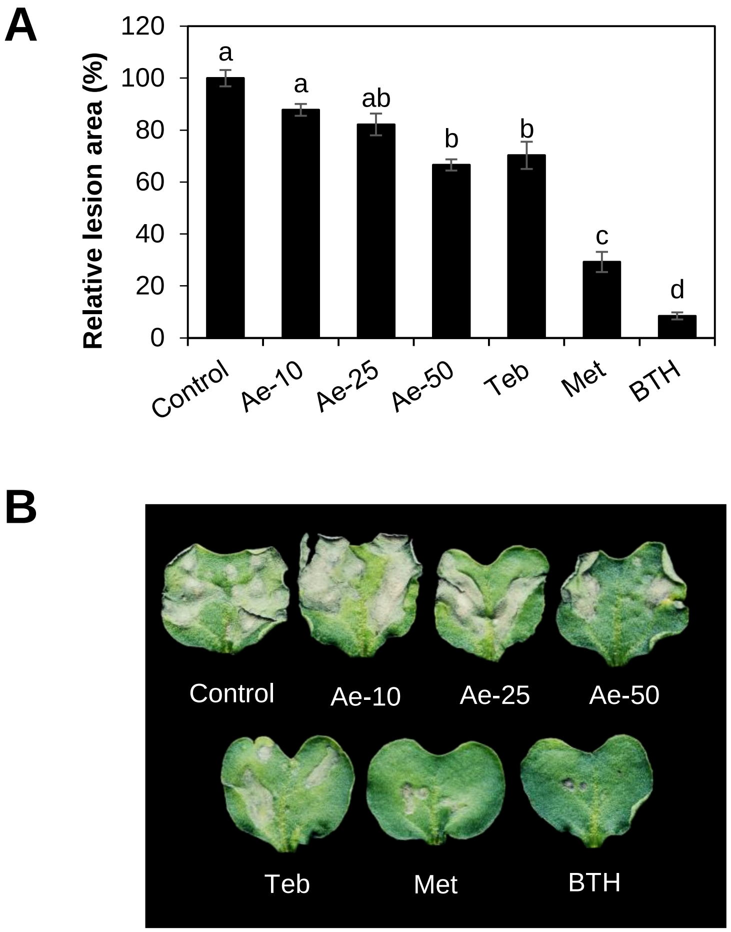

Supplementary Figure 3 | Aescin in spray treatment provides protection to *B. napus* against *L. maculans*. Cotyledons of *B. napus* were sprayed with aqueous solutions of aescin (Ae; at 10, 25, and 50 $\mu g \cdot mL^{-1}$), tebuconazole (Teb; 2 $\mu g \cdot mL^{-1}$), metconazole (Met; 2 $\mu g \cdot mL^{-1}$), BTH (30 μM), or a control 3 days prior to being infiltrated by conidial suspension of *L. maculans* JN2-GFP. The outcome was assessed at 12 days. **(A)** Quantification of the relative lesion area by image analysis expressed in percentages. Control treatment was set as 100%. Data represent means ± SE from three independent experiments. Different letters above bars illustrate significant differences using ANOVA test in conjunction with Tukey's honestly significant difference multiple mean comparison post hoc test ($P < 0.05$). **(B)** A representative leaf for each treatment is shown.

Supplementary Figure 4

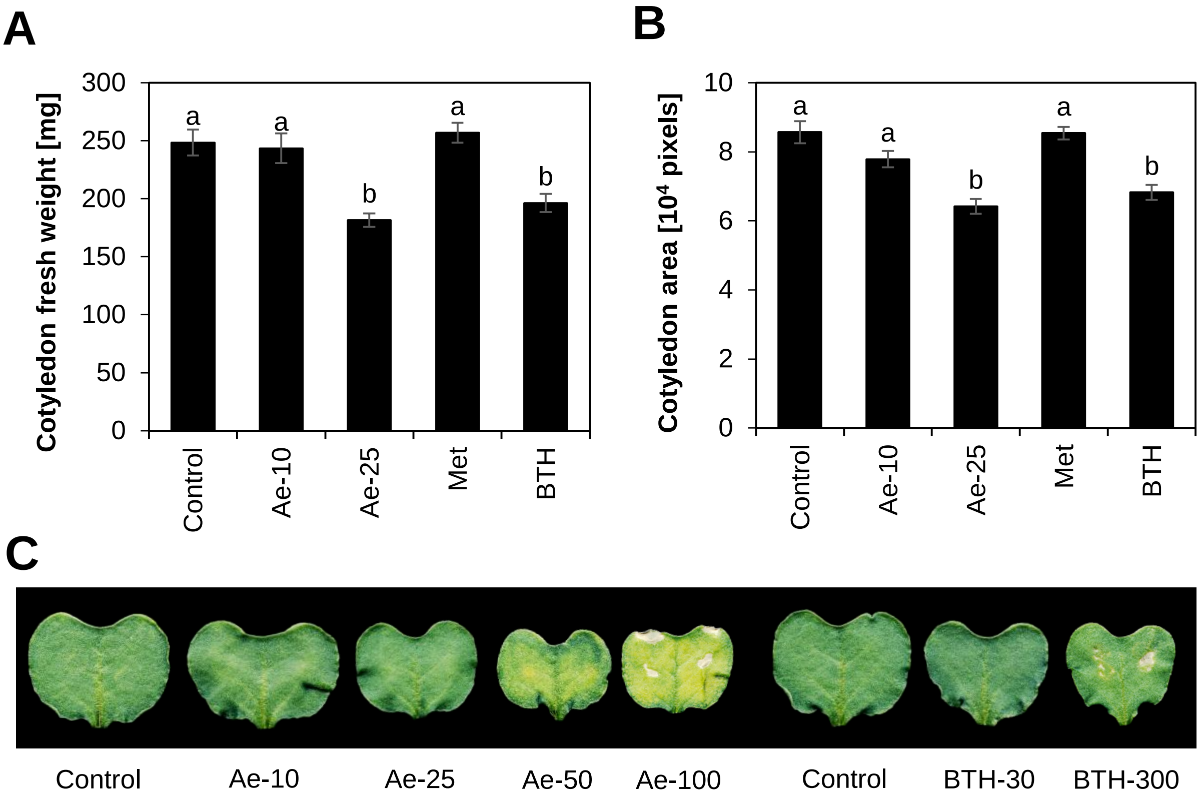

Supplementary Figure 4 | Aescin infiltration treatment reduces plant growth on *B. napus*. Cotyledons of *B. napus* were infiltrated by aescin (Ae; 10 and 25 µg·mL^{-1}), metconazole (Met; 2 µg·mL^{-1}), and BTH (30 µM) and plant growth was analyzed 7 days post treatment. Twelve plants per treatment were used. Data represent means ± SE from three experiments. (**A**) Fresh weight of cotyledons. (**B**) Cotyledon leaf area. Different letters above bars illustrate significant differences using ANOVA test in conjunction with Tukey's honestly significant difference multiple mean comparison post hoc test ($P < 0.05$). (**C**) Panel with representative leaves following aescin (Ae; 10, 25, 50, and 100 µg·mL^{-1}) and BTH (30 and 300 µM) treatment.

Supplementary Figure 5

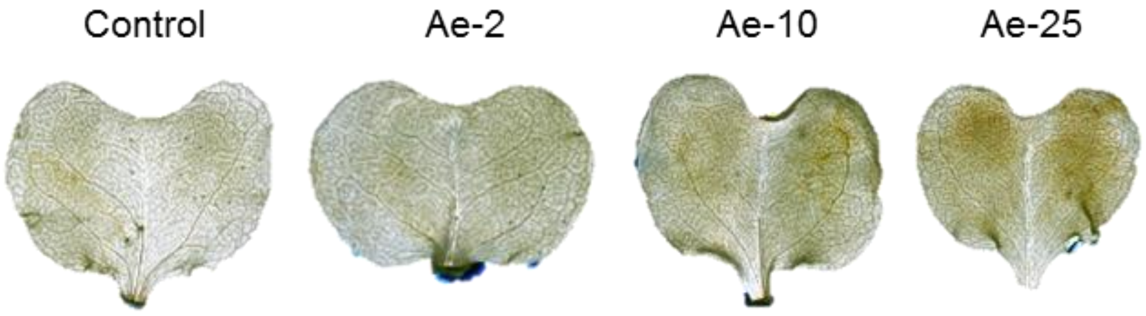

Supplementary Figure 5 | Aescin treatment triggers dose-dependent oxidative burst in *B. napus*. Cotyledons of *B. napus* were infiltrated by aqueous solutions of aescin (Ae; 2, 10, and 25 $\mu g \cdot mL^{-1}$), flg22 (1 µM), or a control treatment. Accumulation of reactive oxygen species visualized by DAB staining at 24 h post treatment. Images are representative of three experiments.

Supplementary Figure 6

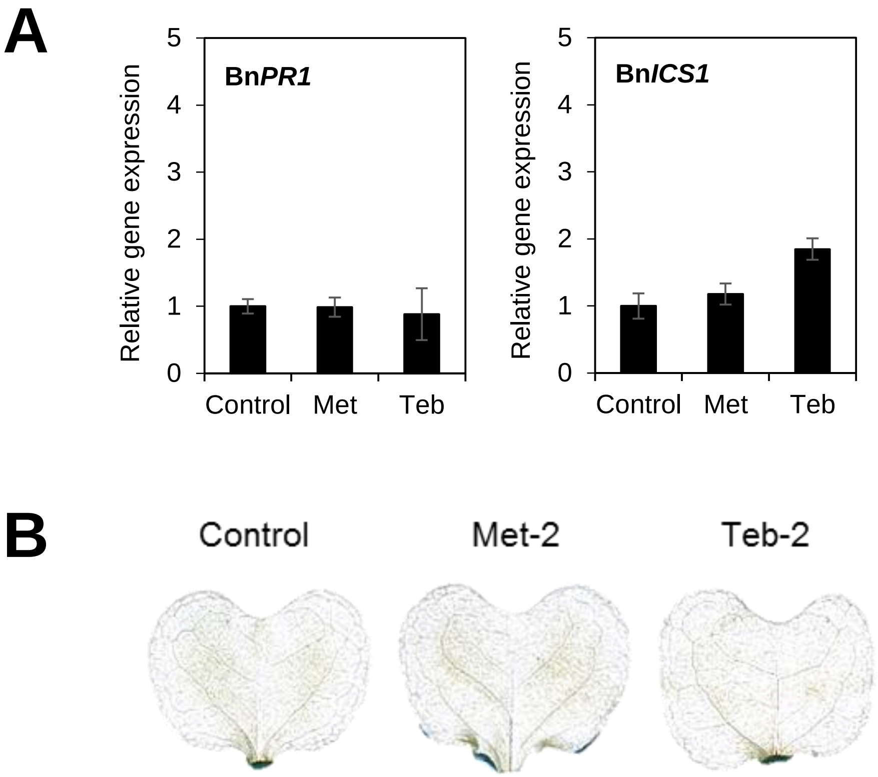

Supplementary Figure 6 | Fungicides trigger neither defense gene transcription nor oxidative burst in *B. napus*. Cotyledons of *B. napus* were infiltrated by metconazole (Met) and tebuconazole (Teb), both at 2 µg·mL^{-1}, then analyzed 24 h later. **(A)** Transcription of *pathogenesis-related BnPR1* and *isochorismate-synthase 1 BnICS1* genes was analyzed by qPCR, normalized to *BnActin*, then compared to the control. Data are from one biological experiment (four biological replicates) representative of three. Data represent means ± SE. **(B)** Accumulation of reactive oxygen species visualized by DAB staining. Images are representative of two experiments.

Supplementary Figure 7

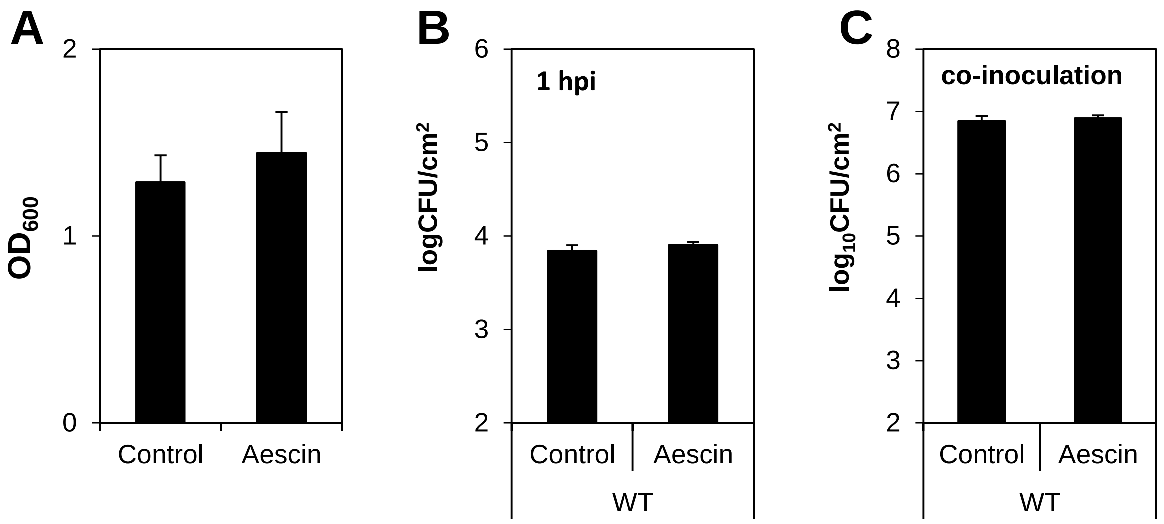

Supplementary Figure 7 | Aescin does not directly impact the growth of *P. syringae* pv. tomato DC3000 (*Pst* DC3000). (A) Growth of *Pst* DC3000 in LB medium after 24 h in the presence of aescin at the 10 µg·mL^{-1} rate or of ethanol (0.1 % (v/v); control) assessed as optical density. **(B)** Bacterial titers of *Pst* DC3000 in *A. thaliana* plants (Col-0; WT) infected after 24 h of pretreatment with aescin at the 10 µg·mL^{-1} rate or of ethanol (0.1 % (v/v); control; 0 *dpi*). Titers were sampled at 1 h post infection. **(C)** Bacterial titers in *A. thaliana* plants (Col-0; WT) at 3 *dpi* after treatment with the joint-solution of aescin at the 10 µg·mL^{-1} rate and *Pst* DC3000 suspension (co-inoculation). The values represent means ± SE from four (A) and three (B, C) independent experiments.

Supplementary Table 1. List of primers used for qPCR.

Gene	Acession n°		Sequence (5' to 3')	Reference
BnActin	XM_013858992	F	CTGGAATTGCTGACCGTATGAG	Šašek et al., 2012b
		R	TGTTGGAAAGTGCTGAGGGA	
BnTIP41	XM_013795927	F	AGAGTCATGCCAAGTTCATGGTT	Chen et al., 2010*
		R	CCTCATAAGCACACCATCAACTCTAA	
BnPR1	XM_013881247	F	CATCCCTCGAAAGCTCAAGAC	Šašek et al., 2012b
		R	CCACTGCACGGGACCTAC	
BnICS1	XM_013827053	F	CAAACTCATCATCTTCCCTC	Šašek et al., 2012b
		R	AGCGTGACTTACTAACCAG	
BnWRKY70	XM_013807970	F	ACATACATAGGAAACCACACG	Šašek et al., 2012b
		R	ACTTGGACTATCTTCAGAATGC	
BnRbohD	XM_013788801	F	TATCCTCAAGGACATCATCAG	this study
		R	TTTCCTCGTCCTAAACCCT	
BnRbohF	XM_013806091	F	TGTTCTCTTATTGGTTGGTC	Šašek et al., 2012b
		R	TTCCTGTGCTGTTCTCTG	
AtSAND	AT2G28390	F	CTGTCTTCTCATCTCTTGTC	Šašek et al., 2014*
		R	TCTTGCAATATGGTTCCTG	
AtTIP41	AT4G34270	F	GTGAAAACTGTTGGAGAGAAGCAA	Leontovyčová et al., 2019
		R	TCAACTGGATACCCTTTCGCA	
AtPR1	AT2G14610	F	AGTTGTTTGGAGAAAGTCAG	Šašek et al., 2014*
		R	GTTCACATAATTCCCACGA	
AtICS1	AT1G74710	F	GCAAGAATCATGTTCCTACC	Šašek et al., 2014*
		R	AATTATCCTGCTGTTACGAG	
LmTUB	XM_003836006	F	TCAAGATGTCCTCCACCT	Šašek et al., 2012b
		R	GTACCAATGCAAGAAAGCC	
LmERG3	XM_003844296	F	ATGGCTCTTCGGTGTCTG	this study
		R	GTGTGGCTTGTGGATGTG	
LmERG11	XM_003840513	F	GATTTACTCGCCACTTACCAC	this study
		R	TGACAAACTTCTTCTGCTCCA	

* Bibliography

Chen X, Truksa M, Shah S, Weselake RJ. (2010) A survey of quantitative real-time polymerase chain reaction internal reference genes for expression studies in *Brassica napus*. *Anal Biochem.* 405(1):138-40. doi: 10.1016/j.ab.2010.05.032

Šašek V, Janda M, Delage E, Puyaubert J, Guivarc'h A, López Maseda E, Dobrev PI, Caius J, Bóka K, Valentová O, Burketová L, Zachowski A, Ruelland E. (2014) Constitutive salicylic acid accumulation in pi4kIIIβ1β2 Arabidopsis plants stunts rosette but not root growth. *New Phytol.* 203(3):805-16. doi: 10.1111/nph.12822
